# Hypothalamic neuropeptides and neurocircuitries in Prader Willi syndrome

**DOI:** 10.1111/jne.12994

**Published:** 2021-06-22

**Authors:** Felipe Correa‐da‐Silva, Eric Fliers, Dick F. Swaab, Chun‐Xia Yi

**Affiliations:** ^1^ Department of Endocrinology and Metabolism Amsterdam Gastroenterology Endocrinology and Metabolism Amsterdam University Medical Center (UMC) University of Amsterdam Amsterdam The Netherlands; ^2^ Laboratory of Endocrinology Amsterdam University Medical Center (UMC) University of Amsterdam Amsterdam The Netherlands; ^3^ Department of Neuropsychiatric Disorders Netherlands Institute for Neuroscience An Institute of the Royal Netherlands Academy of Arts and Sciences Amsterdam The Netherlands

**Keywords:** hypothalamus, microglia, neuropeptides, obesity, Prader‐Willi Syndrome

## Abstract

Prader‐Willi Syndrome (PWS) is a rare and incurable congenital neurodevelopmental disorder, resulting from the absence of expression of a group of genes on the paternally acquired chromosome 15q11‐q13. Phenotypical characteristics of PWS include infantile hypotonia, short stature, incomplete pubertal development, hyperphagia and morbid obesity. Hypothalamic dysfunction in controlling body weight and food intake is a hallmark of PWS. Neuroimaging studies have demonstrated that PWS subjects have abnormal neurocircuitry engaged in the hedonic and physiological control of feeding behavior. This is translated into diminished production of hypothalamic effector peptides which are responsible for the coordination of energy homeostasis and satiety. So far, studies with animal models for PWS and with human post‐mortem hypothalamic specimens demonstrated changes particularly in the infundibular and the paraventricular nuclei of the hypothalamus, both in orexigenic and anorexigenic neural populations. Moreover, many PWS patients have a severe endocrine dysfunction, e.g. central hypogonadism and/or growth hormone deficiency, which may contribute to the development of increased fat mass, especially if left untreated. Additionally, the role of non‐neuronal cells, such as astrocytes and microglia in the hypothalamic dysregulation in PWS is yet to be determined. Notably, microglial activation is persistently present in non‐genetic obesity. To what extent microglia, and other glial cells, are affected in PWS is poorly understood. The elucidation of the hypothalamic dysfunction in PWS could prove to be a key feature of rational therapeutic management in this syndrome. This review aims to examine the evidence for hypothalamic dysfunction, both at the neuropeptidergic and circuitry levels, and its correlation with the pathophysiology of PWS.

## Graphical abstract

Prader‐Willy Syndrome is a genetic obesity syndrome characterized by severe hyperphagia. Here, we provided an overview of defective hypothalamic neurocircuitries and peptides that underlie the gross obesity found in this disorder.

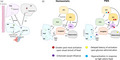

## 1 INTRODUCTION

Prader‐Willi Syndrome (PWS) is a rare congenital neurodevelopmental disorder caused by the loss of genes and non‐coding RNAs on chromosome 15q11‐q13.^1^ The phenotypic features affiliated with PWS are due to a multifactorial disruption of homeostatic processes at cellular and tissue level. Many features are present from a young age; motoric and linguistic milestones are typically delayed, and all individuals have a certain degree of mental retardation.^1^, ^2^, ^3^ In addition, PWS patients suffer from multiple endocrine abnormalities. PWS infants present with hypotonia which, among others, causes an impaired feeding behaviour due to poor suck and swallowing. By contrast, children and adults are characterised by gross hyperphagia and poor satiety, which lead to a severe obese phenotype.^1^, ^2^ The estimated prevalence of PWS is 1/10 000‐1/30 000 cases worldwide. Well‐defined, widely accepted diagnostic criteria are available and can strengthen the suspicion as early as in the foetal period; however, genetic testing remains the pillar of PWS diagnosis currently.^2^ Clinical studies have provided substantial evidence for hypothalamic dysfunction in PWS like uncontrollable hunger^4^, ^5^ and impaired growth^6^, ^7^ and sexual development.^8^ However, the current shred of evidence cannot pinpoint if the disorder is primarily due to hypothalamic defects or the disruptive hypothalamic function is a consequence of imbalance elsewhere. Further, the molecular mechanisms behind hypothalamic dysfunction in PWS are yet to be determined. In this review, we aim to discuss the evidence for hypothalamic dysfunction and the correlation with the clinical and pathophysiological aspects of PWS.

## 2 THE GENETICS OF PWS

**FIGURE 1:**
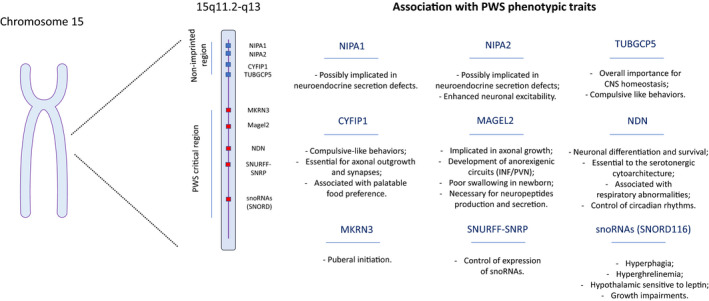
Schematic expression map of the PWS genomic region. PWS is caused by loss of expression of paternally inherited genes located in chrmosome 15. The extension of the deletion is critical for the severity of the phenotype, and patients that lack expression of genes in the non‐imprinted region are reported to present more serious symptons. In addition, the contribuition of the PWS‐causative genes to the phenotypic traits of the disease is given

The PWS phenotype results from the loss of paternally expressed components on chromosome 15q11‐q13. The same genes and non‐coding RNAs derived from the mother are inactivated by imprinting; and thus, not expressed under normal conditions. So far, it is impossible to attribute the phenotypic traits to a single gene; but rather, the symptomology of PWS is a consequence of the entire deletion. In this section, we will briefly discuss our current understanding of the role of each gene and its connections with the disruption of hypothalamic function. In addition, a schematic representation of the expression map of chromosome 15 can be found in Figure 1. Importantly, PWS‐associated loss of expression can be extended to a non‐imprinted region, resulting in a more severe phenotype.^9^ Conventionally, the extension of the deletion led to subdivision of the genotypes. Namely, PWS T1 genotype refers to those that lack expression of both, the critical and non‐imprinted region; whereas PWS T2 is associated with deletion exclusively of the critical region.^9^

### 2.1 NIPA1

NIPA1 encodes a transmembrane protein recruited upon fluctuations in intracellular magnesium concentrations.^10^, ^11^ There is no evidence for the participation of this gene in energy homeostasis in the hypothalamus or periphery. Its localization with endosomes suggests a role in the secretion pathways.^10^ A recent report has demonstrated that neurons from PWS patients have decreased secretory granules and neuropeptides production. However, the authors attribute this defect to the MAGEL2 gene.^12^ Since PWS T1 have more severe phenotypic traits it is possible that the additional loss of NIPA1 hampers the neuroendocrine maturation and secretion.^12^

### 2.2 NIPA2

NIPA2 shares structural and functional similarities with NIPA1. It is also a transmembrane protein sensitive to magnesium fluctuations.^13^ Likewise, there are no in‐depth studies that pinpoints a role for this protein in hypothalamic control of metabolism. However, some reports suggest that NIPA2 has a role in neuronal homeostasis, and thus its deletion might impact optimal neuronal functioning. Maternal protein restriction leads to increased expression of NIPA2 in the foetal hypothalamus in rats,^14^ but the implications of these findings are yet to be clarified. The authors also report an enrichment of mitochondrial metabolism proteins, which reinforces the hypothesis of the participation of NIPA2 in mitophagy.^15^ Further, NIPA2 absence has been linked to increased neuronal excitability in the cortex.^16^

### 2.3 CYFIP1

CYFIP1 is involved in cytoskeleton regulation and neuronal development, especially axonal outgrowth.^17^ It is particularly enriched in synaptosomes,^18^, ^19^ which reinforces the idea of a neuropeptidergic imbalance in PWS. CYFIP haploinsufficiency is also linked with compulsive‐like behaviour and it can be connected with palatable food preference.^20^ Therefore, CYFIP1 expression may relate to PWS symptomatology. In addition to neuronal physiology, CYFIP1 impacts myelination as well, through disruptions of oligodendrocyte biology.^21^

### 2.4 TUBGCP5

This protein encoding gene has a notorious role in centrosome formation,^22^ and therefore impacts the normal process of cellular division. Thus far, it has not been particularly implicated in hypothalamic circuits. Remarkably, disruption of this protein is implicated in microencephaly^22^; indicating an important role in brain development. Similar to CYFIP1, dysfunction or absence of TUBGCP5 is associated with the promotion of compulsive behaviours in neuropsychiatric disorders, such as the autism spectrum disorder.^23^

### 2.5 MKRN3

The Makoring RING protein 3 (MKRN3) is a protein encoding allele, and its deletion is common to all PWS sub genotypes. Here, it is important to highlight the fact that a paternal deletion of MKRN3, MAGEL2 and NDN (the last two will be discussed in sequence) does not result in PWS.^24^ This study reports two PWS subjects with PWS‐like features, but who do not display the core of PWS deletion. One of them, which lacks expression of the three mentioned genes had cognitive impairments and obesity,^24^ suggesting that those genes have a potential role in the phenotypic traits explored in this review.

MRKN3 in the hypothalamus is distinctly involved in the initiation of puberty.^25^ Hypothalamic MKRN3 is highly expressed in early life, both in rodents and nonhuman primates’ restraining signals of pubertal initiation. With the onset of puberty, its expression gradually decreases, which allows the expression of kisspeptin. When untimely released, this leads to gonadotropin‐releasing hormone (GnRH) secretion and sexual development.^25^, ^26^ Loss‐of function mutations in MRNK3 were consistently linked with central precocious puberty (CPP).^27^, ^28^ This is specially intriguing, since CPP is extremely rare in PWS. As far we were able to trace, there are five confirmed cases of CPP in genetically‐confirmed PWS patients^29^, ^30^, ^31^, ^32^ and the exact mechanism behind this phenomenon is still poorly understood. Unquestionably, central control of reproduction is complex and goes beyond the MRNK3‐Kisspeptin‐GnRH axis and even hormones classically associated with metabolism have major reproductive controlling roles.^33^, ^34^ Indeed, although evidence is still very fragmentary for any conclusions in PWS biology the marked hyperghrelinemia (discussed further down) can be a key point to understanding this puzzle. A variety of studies demonstrate a suppressive role of ghrelin in the hypothalamic‐pituitary‐gonadal axis both in vitro and in vivo^35^, ^36^, ^37^ and initial hints indicate that in humans a similar pattern is found.^38^, ^39^

### 2.6 MAGEL2

Melanoma antigen L2 (MAGEL2) is perhaps one of the most intensively explored genes among the PWS deletion. Its cellular function is to facilitate endosomal membrane protein recycling.^40^ It is expressed throughout the central nervous system (CNS), and abundantly present in the hypothalamus.^41^ Studies in MAGEL2 null mice have been insightful and many of the PWS features can be recapitulated in this model. For instance, impaired axonal growth and development of anorexigenic hypothalamic neuronal populations (pro‐opiomelanocortin and oxytocin related circuitry).^42^, ^43^ At the cellular level, its loss results in a decreased neuropeptide production and secretory capacity.^12^ Moreover, MAGEL2 null new born mice also display poor sucking as PWS infants.^44^

### 2.7 NDN

Necdin (NDN) is a nuclear protein exclusively expressed in differentiated neurons.^45^ This protein is mainly implicated with the molecular identity of neuronal cells^45^ and their survival.^46^, ^47^ The understanding of the spatial and temporal events that underlie neuronal dysfunction in PWS pathology is very limited. Interestingly, among all PWS‐related animal models only those with NDN deletion recapitulate the respiratory defects found in PWS‐patients^48^, ^49^ (i.e. sleep apnea).^50^ Recent findings demonstrate that NDN deletion disturbs the serotonergic cytoarchitecture leading to abnormal respiratory patterns.^51^ Lastly, in the murine hypothalamus the NDN transcript is abundantly present in the superchiasmatic nuclei, the master regulator of circadian rhythms.^52^ NDN presence controls the expression of key clock genes and therefore largely impacts the circadian regulation. From the metabolic perspective, these findings have several implications. Circadian integration of metabolism optimizes energy consumption and expenditure across the light/dark circles. Disruption of these patterns are tightly associated with obesity and its comorbidities, such as cardiovascular diseases^53^ and diabetes.^54^

### 2.8 SNUFF‐SNRPN

Little is known about the contribution of this protein to the pathophysiology of PWS. This is perhaps the least explored protein encoding gene in the PWS critical genomic region. However, a report from Cao and colleagues demonstrates that a deletion that causes loss of function of SNUFF‐SNRPN is sufficient to promote PWS‐like symptoms.^55^ The authors speculate this is because SNUFF‐SNRPN is responsible for the expression of key small nucleoar RNAs (sno RNAs – discussed next), that are central factors in the PWS symptomatology traits.^55^

### 2.9 sno RNAs

In addition to the protein‐coding alleles, non‐coding RNAs are found in the critical genomic region of PWS, especially snoRNAs. The exact biology of these RNAs is yet to be defined, but it has been proposed that they are involved in the modification of other RNAs.^56^ One special cluster of these biomolecules – the Snord116‐ is highly implicated in PWS and has gained crescent attention. Snord116del mice are of special interest in PWS research, because those mice in which the snoRNA116 deletion is of paternal heritage display hyperphagia.^57^ However, the Snor116del mice do not show increased body weight, even when fed an obesogenic diet. In fact, these mice present characteristically reduced body weight, delayed sexual maturation and high rates of mortality prior to weaning. The hypothalamic dysfunction associated with Snord116del can be explained by endocrine imbalance and sensitivity to adipostatic signals. These will be discussed into more detail in the sections below.

Taken together, it is possible to conclude that none of the individual features of the syndrome can be attributed to any single causative gene. Rather, the PWS symptomatology is a direct result of the cumulative deletion. Although animal models allowed us to have insights into the participation of each gene in the phenotype, we are far from understanding PWS at the molecular level. Certainly, the rarity of the syndrome and the limitations associated with human studies are the main sources of obstacles currently.

## 3 HYPOTHALAMIC NEUROCIRCUITS IN PWS

**FIGURE 2:**
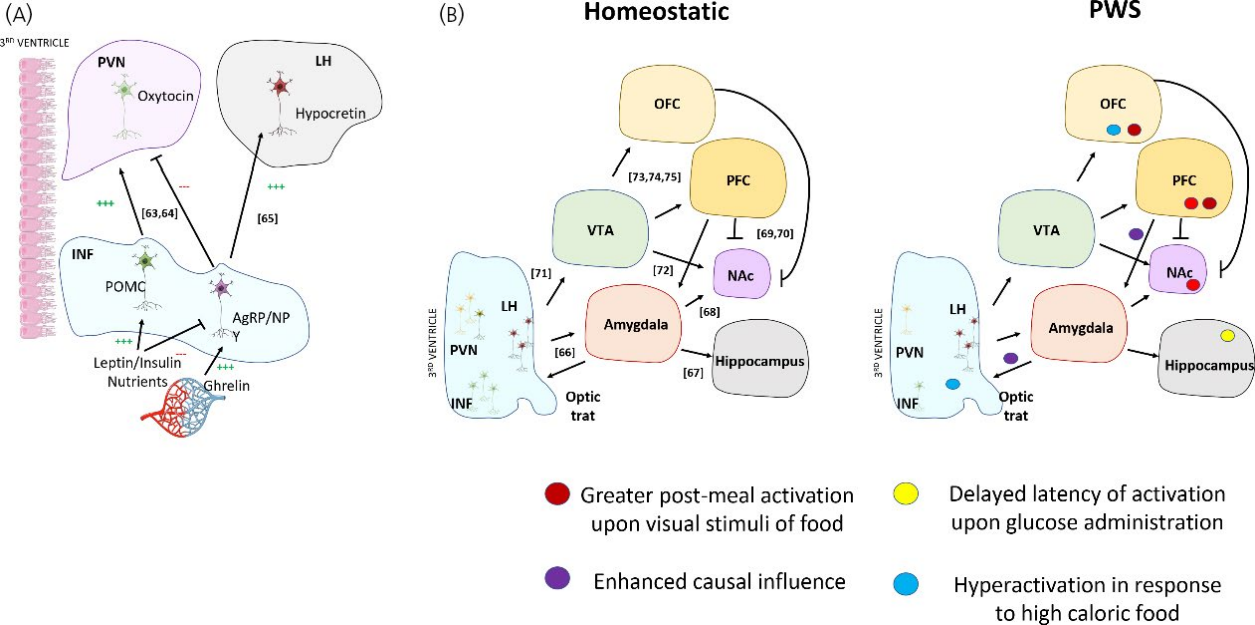
Schematic representation of the neurocircuitry mediating hunger and hedonic components of feeding. Diagrammatic representation of a coronal hypothalamic human section highlighting the infundibular nucleus (INF), paraventricular nucleus of hypothalamus (PVN) and lateral hypothalamus (LH) neuronal populations responsible for the homeostatic control of energy homeostasis. In brief, peripheral factors (hormones and nutrients) activate INF neurons, which will propagate the orexigenic/anorexigenic response throughout the hypothalamus generating autonomic and neuroendocrine outputs consistent with primary signal^247^ (A). Schematic overview of the hypothalamic and cortical (hedonic) components responsible with feeding. Electrical stimulation of LH neurons leads to inputs in cortical structures involved with behavior choices of feeding and reward centers. The combination of the homeostatic drive of feeding, learned behavior and hedonic components constitute the choice of eating.^74^ Next, representation of the known disruptions of this neurocircuitry in PWS (B). INF, Infundibular nucleus; LH, lateral hypothalamus; NAc, nucleus accumbens; OFC, orbitofrontal cortex; PFC, prefrontal cortex; PVN, paraventricular nucleus; PWS, Prader Willi Syndrome; VTA, ventral tegmental area

The insatiable hunger experienced by PWS subjects is strongly indicating a malfunction of the hypothalamic control of feeding behaviour. Functional magnetic resonance imaging (fMRI) studies of the hypothalamus and cortex have given a better understanding of satiety‐related events in PWS. PWS patients display abnormal brain networks, engaged with physiological control of eating and the motivational component of feeding.^58^, ^59^, ^60^, ^61^ Holsen *et al* ^58^ exposed healthy controls and PWS participants to visual food stimuli in a pre‐ and post‐meal condition and evaluated the activation of neuronal networks related to food motivation. Interestingly, PWS patients presented greater cortical activation to food stimuli in a post‐meal state. By contrast, healthy subjects presented an opposite pattern, with stronger function in the pre‐meal state.^58^ Consistently, upon glucose consumption PWS subjects have delayed satiety‐associated neural circuit activation in the hypothalamus and extra hypothalamic areas.^59^ These findings suggest that perception of nutritional status is delayed in PWS patients, likely leading to a defective hypothalamic response to nutrients. Interestingly, it has been shown that the hyperfunction of those neuronal networks is particularly associated with high caloric foods rather than low caloric stimuli.^61^ A schematic overview of the neurocircuitry engaged in feeding at the hypothalamic^62^, ^63^, ^64^ and cortical^65^, ^66^, ^67^, ^68^, ^69^, ^70^, ^71^, ^72^, ^73^, ^74^ levels can be found in Figure 2.

Undoubtedly, neurocircuitries in the brain of patients with PWS are already malfunctioning in the early life stage. This idea is supported by the study of Zhang *et al* who found alterations in the baseline brain activity of PWS children compared to control siblings. In this study, a combination of fMRI with causality analysis was employed to understand the behavioural and mechanistic components of hyperphagia in PWS. The study showed significantly increased impacts of amygdala onto the hypothalamus. Moreover, cortical influences on the amygdala were also increased.^60^ The cortical‐amygdala‐hypothalamic axis is implicated in the coupling of the behavioural component of feeding with the neuronal populations engaged with initiation or termination of eating in the hypothalamus.^75^ The authors suggest that the enhanced cortical inputs into the amygdala play a crucial role in an imbalanced cognitive processing, which can lead to an increased eating motivation in PWS. The combination of abnormal cortical inputs, and increased influence onto hypothalamic circuits can lead to the insatiable state of hunger found in PWS patients. Those findings are especially interesting when the hyperstimulation of the hypothalamic circuits takes place in the baseline condition, meaning that a hunger state is evoked regardless of any food stimuli (i.e. visual).^60^

The hypothalamus is a highly heterogeneously structured area engaged in behavioural, autonomic, and endocrine functions in response to the environment.^76^, ^77^, ^78^ Extensive studies in rodents and humans demonstrated that disruption of different hypothalamic neurocircuitries that control energy homeostasis is underlying the development of metabolic diseases.^79^, ^80^, ^81^, ^82^, ^83^, ^84^ The infundibular nucleus (INF, or arcuate nucleus in rodents) plays a key role in the central management of feeding behaviour and energy expenditure. Under physiological conditions, feeding activates the anorexigenic neuronal population located at the INF, characterized by the expression of pro‐opiomelanocortin (POMC).^85^ Antagonistically, an appetite‐stimulating neuronal population is characterized by the expression of neuropeptide Y (NPY) and agouti related protein (AgRP).^86^ Moreover, the activity of the regulatory neuronal populations associated with energy homeostasis are modulated by humoral feedback from the periphery. Receptors for peripheral metabolic hormones such as insulin, leptin and ghrelin are expressed on POMC and AgRP/NPY neurons.^87^, ^88^ The opposed nature of these neuronal populations creates a counter‐regulatory system of energy homeostasis. The INF neuronal populations project to the paraventricular nucleus of the hypothalamus (PVN),^89^ in which the satiety or hunger signals will be perpetuated through neuroendocrine mediators or (para)sympathetic outflow to peripheral metabolic organs. Arginine vasopressin (AVP)‐ and oxytocin (OXT)‐ expressing neurons are two major neuronal populations located in the PVN and are largely implicated in inhibition of feeding behavior.^90^ Additionally, other neuronal populations that reside in the PVN are implicated in the global metabolic control. Corticotropin‐releasing hormone‐ (CRH) expressing neurons inhibit food intake and promote energy expenditure.^91^ Moreover, CRH neurons interact with leptin‐sensitive inputs from the arcuate nucleus in mice.^92^ Furthermore, thyrotropin‐releasing hormone‐ (TRH) expressing neurons in the PVN co‐express leptin receptors and participate in energy metabolism due to their involvement in the hypothalamus‐pituitary‐thyroid axis impact on thyroid function.^93^ TRH neurons propagate the anorexigenic signal^93^, ^94^ and have a protagonist role in thermoregulation.^95^ Additionally, orexin neurons in the lateral hypothalamus (LH) are involved in feeding‐regulation,^96^, ^97^, ^98^ although they play a less potent role than the NPY and AgRP populations.^99^ In the fasting state, the levels of the precursor peptide of orexin in the hypothalamus are elevated,^100^ whereas central administration of orexin leads to food intake in a dose‐dependent manner.^98^ Moreover, orexin has also been involved in the motivational component of feeding as well.^101^

Likely, the gross obesity of PWS patients has multiple mechanisms that culminate in the disease phenotype. Among those factors, it is possible to highlight the disruption of satiety control,^4^, ^5^, ^58^ endocrine dysregulation (central and peripheral),^6^, ^7^ reduced energy expenditure due to hypotonia and behavioural features of the syndrome. Indeed, studies with post‐mortem hypothalamic tissue specimens have shown that in PWS patients, neurons in a variety of hypothalamic areas are affected, including the INF, PVN and LH.^102^, ^103^

## 4 HYPOTHALAMIC NEUROPEPTIDERGIC SYSTEM IN PWS

Extensive studies in rodents and humans demonstrated that disruption of the hypothalamic neuropeptides engaged in energy homeostasis promote the obese phenotype.^104^, ^105^, ^106^, ^107^ Mutations in the melanocortin 4 receptor (MC4R) are the most common cause of monogenic obesity.^80^, ^108^, ^109^, ^110^ In this case, obesity is a consequence of the lack of POMC‐derived peptide signalling in MC4R‐expressing neurons, resulting in a chronic orexigenic state.^108^, ^109^ A study with post‐mortem human brain tissue also showed that the expression of NPY and AgRP has a close correlation with the individual’s body mass index (BMI).^111^ It is thought‐provoking to address the importance and limitations of immunohistochemistry on post‐mortem tissue, which is the major source of literature on neuropeptides at the protein level in PWS. Due to the rarity of the PWS post‐mortem tissue and the lack of representative animal models that fully recapitulates its phenotype functional interpretation is a challenge. Of notice, hypothalamic post‐mortem material has been extensively used in other fields of research and has proved to be reliable, such as in non‐genetic obesity,^112^ diabetes,^84^ and mood disorders.^113^ However, the analysis of neuropeptides’ immunoreactivity without other parameters or support of the literature requires caution. This is especially due to alterations in the balance of synthesis, maturation, and secretion of these peptides. Increased levels of a neuropeptide can be explained as increased production or a defective or decreased transport and release. In the same way, reduced immunoreactivity can also indicate rapid turnover of the protein and not necessarily decreased production. This problem can be illustrated by the discrepancy between an increased vasopressin content in the SCN together with an diminished vasopressin production in female depressed patients.^114^ Therefore, human post‐mortem material is a reliable and stable source of study, especially in rare pathophysiology such as PWS; however the interpretation of data requires a global overview of the studied systems.

### 4.1 Hypothalamic orexigenic neuropeptides in PWS

NPY neuron numbers are consistently downregulated in obese and PWS‐obese subjects.^103^ At first glance, reduction in NPY cell counts seems counterintuitive since an insatiable hunger is a major feature of PWS.^4^ The molecular mechanisms behind this reduction are yet to be clarified. Diminished orexigenic signalling might be explained by increased plasma levels of adipostatic hormones such as leptin and insulin,^115^, ^116^ responsible for inhibition of neuronal activity of NPY and AgRP neurons. Evidence from murine studies demonstrates a reduction of NPY transcript in the hypothalami of high fat diet‐fed animals.^117^, ^118^, ^119^ It is believed that the chronic exposure of these adipostatic factors leads to malfunctioning and eventual death of the orexigenic neuronal populations.^117^ The role of peripheral endocrine factors in the pathophysiology of PWS will be addressed in a further section.

Interestingly, in the study with post‐mortem hypothalamic tissues, Goldstone *et al* ^103^ reported unchanged expression of AgRP in PWS. The authors found a tendency for reduction of AgRP immunoreactivity in the INF of PWS and non‐genetic obese individuals, which was lost when the values were adjusted according to premorbid illness duration. This finding is consistent with reports of murine models of obesity, in which the AgRP expression is unaltered.^120^ In contrast, a recent study examined the transcriptomic signature of PWS patients and found a 3‐fold upregulation in the AgRP transcript.^121^ Additionally, genes that are overrepresented in the PWS hypothalami overlap with the murine AgRP identity.^121^ The discrepancy between both studies might be explained by a defective post translational processing of AgRP in PWS. The enzyme prohormone convertase PC1 is responsible for the posttranslational cleavage of the AgRP transcript.^122^ Furthermore, PWS patients have reduced levels of prohormone convertase PC2,^123^, ^124^ largely implicated in the posttranslational processing of hypothalamic neuroendocrine mediators.^125^ Interestingly, Burnett *et al* ^126^ found a reduction in PC1 expression in neurons derived from induction of pluripotent stem cells obtained from a PWS patients, compared to those derived from unaffected controls. Thus, it is likely that even though there is an enrichment of an AgRP‐related transcripts in the hypothalami of PWS, due to defective peptide maturation unaltered levels of protein expression are reported. However, a more solid proof of concept on this hypothesis is needed. It is also worth to mention that PC1 is not only responsible for AgRP processing, but takes place in POMC, ghrelin and insulin post translational modifications.^127^ Thus, the infundibular orexigenic neuronal population is hypoactive in PWS, either due to defective secretory mechanisms or (possibly) chronic exposure to peripheral inputs that negatively regulate those populations. Further investigation is necessary to understand this hypoactivity and the underlying mechanisms that allow hyperphagia in this condition.

The LH is a recognized orexigenic centre which receives INF projections.^128^ Neuropeptides produced by neuronal populations in this area, such as hypocretin (orexin) exert appetite‐stimulating functions.^129^ Interestingly, studies on post‐mortem hypothalamic specimens did not find alterations in the distribution and abundance of hypocretin‐expressing neurons in PWS compared to matched controls.^130^ Consistently, hypocretin levels are unaltered in the cerebrospinal fluid of PWS patients. Surprisingly, hypocretin plasma levels are increased in PWS.^131^ It is uncertain to what extent the levels of plasma hypocretin accurately reflect the central presence of this neuropeptide. Collectively, those data indicate increased activity of this neuronal population. Hypocretin receptors are densely expressed throughout the CNS and peripheral tissues^98^, ^132^ Orexin receptors are also found in the human adipose tissue,^133^ and a study has shown that hypocretin signals promote adipose tissue expansion (through the promotion of adipogenesis) and fat deposition in mature adipocytes (through inhibition of lipolysis).^134^ Thus, elevated levels of plasma hypocretin in PWS might contribute to the hypertrophy of adipose tissue in PWS, and consequently, promote weight gain. Interestingly, hypocretin has a role in sleep regulation,^135^, ^136^ which is also markedly disturbed in PWS.^137^ Therefore, the involvement of the hypocretin system in PWS is beyond the regulation of energy metabolism and may play a more complex role in PWS pathophysiology.

An overview of the main findings regarding the neuropeptides explored so far is summarized in Table 1.

**TABLE 1:** Summary of hypothalamic anorexigenic and orexigenic neuropeptides in Prader‐Willi Syndrome

Neuropeptide	CNS	Hypoactivity/Hyperactivity	Plasma concentrations	Reference (##)
NPY	Decreased expression and cell count	Hypoactivity	–	^103^
AgRP	Decreased expression and unchanged cell count	Hypoactivity	–	103, 121
Hypocretin	Unchanged cell count	Unaltered/ Hyperactivity (?)	Increased	130, 131
POMC	Decreased expression	Hypoactivity	–	^121^
OXT	Decreased expression and cell count	Hypoactivity	Increased	102, 142
BDNF	Decreased expression	Hypoactivity	Decreased	121, 146

Abbreviations: AgRP, agouti related protein; BDNF, brain derived neurotrophic factor; NPY, Neuropeptide Y; OXT, oxytocin; POMC, proopiomelanocortin.

### 4.2 Hypothalamic anorexigenic neuropeptides in PWS

Surprisingly, although impaired satiety is a hallmark in PWS,^5^, ^58^ the well‐known anorexigenic POMC has been poorly explored in the disorder. Transcriptomic analysis revealed that, in line with the defective satiety, POMC‐associated genes are downregulated in the hypothalamus of PWS patients.^121^ Animal models have shown that impaired POMC function is central to the development of features of PWS.^42^ This is translated into defective anorexigenic neurocircuitry and impaired leptin sensitivity in this neuronal population.^42^ As PC1/2 expression is diminished in PWS neurons, it is plausible to hypothesize that lower levels of α‐MSH can be found in the hypothalami of PWS patients. Therefore, a marked reduction of expression of α‐MSH and cell number is expected in PWS.

Diminished anorexigenic oxytocin expressing neurons were also observed in the PVN of PWS.^102^ A clearly abnormal neuroanatomy and decreased abundance of oxytocin‐expressing neurons was reported in the PVN of PWS patients which was consistently recapitulated in animal models of PWS.^43^, ^44^ Diminished expression of oxytocin in PWS was confirmed on the RNA level,^121^ corroborating the findings based on immnunostained neuronal counts. Postnatal central administration of this neuropeptide in PWS animal models has been shown to successfully restore metabolic outcomes of the syndrome and promoted positive effects on behavior.^44^ To this date, five clinical trials employed oxytocin in the treatment of PWS.^138^, ^139^, ^140^, ^141^, ^142^ In short, the administration of oxytocin has effects on a behavioural level but is not yet proven to be an effective treatment of all PWS symptoms, and much more evidence is required before it can be used as a therapeutic tool. In contrast with the hypothalamic pattern, plasma oxytocin is reported to be increased in PWS individuals.^143^ Moreover, in concordance with this, increased plasma levels of oxytocin have been reported in PWS children when compared to unaffected siblings.^143^ The discrepancy between the increase in plasma oxytocin levels and the reduction in cell counts and expression at central level exemplify our limited knowledge on the hypothalamic pathophysiology of PWS. Oxytocin has an inactive and an active form, which is not dissociated in the screening of plasma samples.^144^ PWS patients have been reported to have diminished oxytocin receptor expression,^145^ which adds to the level of complexity. Bochukova *et al* ^121^ demonstrated a clear reduction of hypothalamic brain‐derived neurotrophic factor (BDNF) in PWS. The authors propose that the element of neurodegeneration in the pathogenesis of PWS might be associated with this phenomenon. BDNF and its receptor TrKB (encoded by ntrk2) expression were found to be decreased in the ventromedial nucleus (VMH) of the hypothalamus.^121^ Furthermore, plasma levels of BDNF in fasting conditions are decreased in PWS compared to healthy controls.^146^ Notably, beyond its trophic role, BDNF and its receptor have been implicated in suppression of feeding behavior.^147^ Diminished levels of this peptide seem to be consistent with the phenotype observed in PWS, reported to have an unhealthy microenvironment for neuronal populations and satiety deficiencies.^148^, ^149^

An overview of the alterations in neuropeptides can be found in Table 1.

## 5 HYPOTHALAMUS‐REGULATORY METABOLIC HORMONES IN PWS

As discussed so far, the hypothalamic neuronal populations engaged with energy homeostasis are severely affected in PWS. Hypothalamic neuronal malfunction might be directly induced by the genetic defects of PWS but can also be indirectly caused by the neuroendocrine dysregulation which occurs in conjunction with the primary PWS phenotype. Circulating metabolic hormones (i.e. ghrelin, leptin, insulin and adiponectin) inform the hypothalamic neurocircuits about the nutritional status of the organism.^150^ Here we will discuss the current understanding of the role of endocrine factors in the aetiology of PWS.

### 5.1 Ghrelin

Ghrelin is an orexigenic hormone produced by enteroendocrine cells.^151^, ^152^ Central or peripheral administration of ghrelin induces eating and promotes adiposity.^153^, ^154^ In fasted conditions, ghrelin levels are elevated and by contrast, re‐feeding or oral glucose administration reduces the total plasma concentrations of this hormone. Ghrelin acts in the hypothalamus via NPY/AgRP neuron^154^ signalling while POMC‐expressing neurons do not express functional receptors for this hormone.^155^ Genetic ablation of ghrelin or its receptor leads to resistance to diet‐induced obesity in murine models.^156^ Furthermore, hypothalamic resistance to ghrelin signalling is observed upon obesogenic cues.^157^ Intriguingly, ghrelin levels are reduced in human non‐genetic obesity.^158^ Whether humans also have hypothalamic resistance to ghrelin is yet to be determined.

Differently from non‐genetic obesity, ghrelin is found to be upregulated in PWS, and has been implicated as an underlying cause of hyperphagia in PWS.^159^, ^160^, ^161^, ^162^ DelParigi *et al* ^160^ reported a 2.5‐fold increase in PWS plasma levels of ghrelin compared to lean controls; and a 4.5‐fold increase compared to obese subjects. The difference remained significant even after adjustment for percent of body fat.^160^ Ghrelin levels remained elevated in PWS patients in comparison to matched controls even after the consumption of satiating dose liquid meals.^160^ This finding matches with the persistent urge to eat found in PWS. Moreover, plasma ghrelin levels and subjective rating of hunger have a positive correlation in PWS.^160^ It would be interesting to study the ghrelin levels in PWS infants before the onset of hyperphagia. However, no differences were found between non‐obese PWS infants (under the age of 5) and matched controls regarding their ghrelin levels.^163^ In a different study, PWS obese children (average age 9.5 years) had elevated plasma ghrelin.^164^ It is still possible that hyperghrelinemia occurs prior to the onset of obesity.^163^ Nevertheless, Feigerlova *et al* ^161^ also concluded that ghrelin levels are consistently increased in all analysed ages (0‐17 years old).

Currently, no animal model is available that can fully mimic the phenotype of PWS in humans, possibly due to the complex genetic components of the syndrome which complicate the development of reliable animal models.^165^ Yet, there are numerous studies that investigate the role of specific genes present in the PWS critical deletion region. Such PWS mice models are e.g. employed to investigate hyperphagic related features of the syndrome, as in the Snord116del mice.^57^, ^166^ In ad libitum conditions, these mice show increased levels of ghrelin (approximately 2‐fold increase when compared to wild type).^57^ This is the same magnitude of increase expected in wild type mice upon 24 hours fasting. Another study showed Snord116del mice in a ghrelin‐deficient background did not impact the mortality or maturation of sexual traits evoked by the Snord116 loss.^167^ However, the ghrelin‐deficient background promoted leanness and reduction of body fat observed in the single mutant.^167^ Although with divergent phenotypical traits of the PWS pathophysiology (i.e. leanness), this model seems to be particularly interesting to comprehend, at least partially, the hyperphagic state of the syndrome.^168^ It is interesting to note that Snordel116 mice have reduced PC1 expression.^126^ This protein acts in ghrelin posttranslational maturation,^127^ as in POMC.^169^ Although ghrelin levels are elevated in Snord116del mice, there is an increased ratio between the pro‐ghrelin and the mature hormone, due to diminished expression of PC1.^126^ Therefore, concomitantly to the hyperghrelinemia, Snord116del mice are less efficient in ghrelin’s post translational processing. The exact implications of those findings in PWS patients are yet to be determined.

It is worth to notice that extra hypothalamic actions of ghrelin can promote hunger/eating behaviour. The ghrelin receptor is expressed in dopaminergic neurons in the ventral tegmental area (VTA),^170^ and local injection of the peptide in the VTA evokes feeding in a dose dependent manner.^171^ Ghrelin administration increases dopamine turnover in the mesolimbic system upon food consumption, suggesting a role of it in the hedonic component of eating,^172^, ^173^ at least in rodents. Yet, it is clear that the impact of ghrelin in extra‐hypothalamic areas remains to be clarified both in rodents and humans.

In summary, there is no solid evidence for a relation between hyperghrelinemia and hypothalamic dysfunction in PWS. The lack of experimental models and limited human‐derived samples limits our knowledge of ghrelin`s role in the induction of hyperphagia in PWS. In addition, if hyperghrelinemia is the major cause of the drastic hyperphagic state in PWS it is undetermined until now what the main neuronal population behind this orchestration is. This phenotype might be due to ghrelin`s actions on hypothalamic orexigenic neurons, mesolimbic neurons that have direct inputs into the hypothalamus or a synergic effect among them.

### 5.2 Leptin

Leptin is an adipokine that regulates energy homeostasis by signalling hypothalamic centers.^115^ Leptin is a potent anorexigenic hormone, and it is well recognized for activation of POMC‐expression neurons while suppressing the NPY and AgRP neuronal activity.^85^ Leptin also exerts its functions through glial cells.^89^, ^174^ Recently the importance of leptin receptors in both astrocytes^174^ and microglia^89^ has been demonstrated. It is well‐known that lack of leptin leads to a severe obese phenotype and deletion of its receptor also leads to obesity and diabetic traits.^175^ Moreover, central resistance to leptin signalling is a hallmark of obesity in animal models and human subjects.^176^, ^177^

The participation of leptin in the clinical features of PWS is less clear than what is known for ghrelin. Lindgren *et al* ^178^ demonstrated that leptin expression is increased in the adipose tissue of PWS children. This is paralleled by increased plasma levels of leptin in PWS infants.^178^ In agreement with these findings, Butler *et al* ^179^ reported that obese individuals have higher leptin levels compared to their lean counterparts. However, in the lean group, women presented with higher plasma levels of leptin, which was not observed within the PWS population. No sex differences were observed among non‐obese PWS individuals. Furthermore, the only difference in leptin plasma levels found between control and PWS subjects is between lean males and non‐obese PWS males. The authors proposed that this difference might be related to hypogonadism, characteristic of PWS males.^179^ Of note, leptin is a major regulator of reproduction.^180^ Goldstone *et al* ^181^ also did not find differences in plasma leptin between lean and PWS adult women. These differences on plasma levels of leptin cannot be explained by defective secretion or defects in functionality of the receptor.^181^

Snord116del mice have unaltered leptin levels.^168^ Hypothalamic genes associated with leptin signalling genes also remain unchanged in these mutants compared to their wild type littermates.^168^ Interestingly, adenovirus‐mediated deletion of Snord116 in the hypothalamus leads to increased expression of the suppressor of cytokine signalling 3 (SOCS3) in obese animals.^168^ This gene is responsible for the suppression of leptin signaling.^182^ However, the cited work cannot dissociate the contribution of the obese phenotype versus the deletion itself in the elevated SOCS3 expression.^168^ Another animal model that is often employed in PWS research is the MAGEL2 null mouse.^183^ This knockout is insensitive to the anorexigenic effects of leptin,^184^ hence POMC neurons fail to be activated upon leptin exposure. However, whether this is a congenital defect is still up for debate. Moreover, POMC neuron fibers that project to the PVN are reduced in the MAGEL2 mutant mice.^42^

### 5.3 Insulin

Insulin is a hormone secreted by β‐cells of the pancreatic islets. Molecular resistance to insulin or plasma insufficiency leads to the development of type 2 diabetes mellitus (T2DM).^185^ Hypothalamic insulin action takes place in synergism with leptin signalling, and therefore has also a potent anorexigenic effect.^186^ The morbid obesity in PWS is believed to be the main cause of T2DM in PWS population. Indeed, the prevalence of T2DM is much higher in PWS as compared to the general population. A report from Vanderpump shows a prevalence of approximately 3 to 6 percent of T2DM in an English cohort.^187^ By contrast, concomitant studies reported that approximately 25% of PWS individuals are diabetic in other European cohorts,^188^, ^189^ and even a higher percentage is suggested in an Asian population, with approximately 30% of T2DM incidence in PWS.^190^ Despite the clear incidence of T2DM within PWS population, there are discrepant reports in the literature on the role of defective glucose‐regulation in the pathophysiology of PWS. Furthermore, increased plasma concentrations of additional metabolism‐controlling hormones (i.e. ghrelin and adiponectin)^191^ are thought to have great influence in glycaemic control in PWS pathophysiology.

Butler *et al* ^192^ determined that fasting glucose and insulin levels were comparable between PWS and non‐syndromic obese individuals. PWS patients were reported to have significantly higher concentrations of glucose and insulin compared to lean individuals.^192^ Moreover, PWS patients were reported to have hyperinsulinemia response to an oral glucose tolerance test (OGTT) when compared to normal weight controls,^193^ while obese non‐PWS controls displayed comparable OGTT readout to PWS individuals in this study.^193^ In another study, an intravenous glucose tolerance test (IGVTT) showed comparable glucose assimilation coefficient between PWS and non‐syndromic obese population.^194^ These data on glucose assimilation and insulin concentration upon oral glucose challenge indicate that the insulin sensitivity among non‐syndromic obese and PWS should also be comparable. Interestingly, after a protein meal ingestion, PWS patients showed a similar insulin peak to healthy weighted controls, whereas obese non‐PWS controls had a clear insulin peak upon the meal consumption.^195^

Schuster *et al* ^196^ demonstrated that PWS infants have lower insulin response to oral glucose test compared to BMI and age matched individuals. In the same study, no differences were found in glucose or insulin levels on PWS adults when compared to lean or obese controls. The authors discuss that the PWS limited group size might influence the interpretation of this data. Interestingly, on OGTT, PWS presented delayed peak of glucose and insulin when compared to obese controls. It was proposed that this difference might be explained by reduced pancreatic β‐cell responsiveness to glucose fluctuations in PWS population. Consistently with previous literature, the authors found no differences in glucose assimilation assessed by IVGTT.^194^ However, insulin and peptide C plasma levels were reduced in PWS.^196^ This led to reduced insulin to glucose ratios during IGVTT in PWS compared to obese control group. Furthermore, the authors were the first to demonstrate increased hepatic insulin extraction and insulin clearance in PWS compared to obese controls.

A recent study described a heightened fasting insulin levels and sensitivity in PWS infants compared to BMI and age‐matched controls.^197^ However, this study lacks the comparison between PWS and lean subjects. Therefore, it is impossible to determine if in this cohort, whether PWS are normoinsulinemic during fasting. In concordance with the insulin sensitivity data, a more recent report described that PWS individuals have lower fasting insulin levels when compared to obese controls. Not only insulin concentrations were lessened, but also PWS individuals were also proven to have greater insulin sensitivity, which were compared to lean controls.^191^ Thus, although the prevalence of T2DM is greater in PWS, an unexpected increase in insulin sensitivity is found within this population as well. The link between insulin resistance and T2DM is long recognized.^198^ In addition, molecular resistance to insulin signalling is one of the most accurate predictors of development of T2DM and the main therapeutic target for this disorder.^199^, ^200^ There are potential mechanisms that are used to explain this paradoxical clinical feature of PWS. Firstly, although PWS patients display abnormal fat deposition, that occurs preferentially in subcutaneous depots rather in visceral ones.^191^, ^201^, ^202^, ^203^ The visceral fat deposition, which is observed in obese controls, has a positive correlation with lowered sensitivity to insulin signalling.^204^ Moreover, it is known that PWS patients are deficient in growth hormone.^6^, ^205^ This hormone has glucoregulatory roles and, it is particularly interesting to pinpoint that there is a transient induction of insulin resistance in puberal development due to elevated secretion of growth hormone.^206^ Lastly, increased insulin sensitivity in PWS population can eventually be explained by increased levels of adiponectin.^202^ This is an adipose tissue‐derived hormone and targets insulin‐producing cells in the pancreas.^207^ Adiponectin is associated with increased insulin sensitivity and has anti‐inflammatory properties.^208^ Compared to non‐syndromic obese population, PWS individuals have higher plasma concentrations of this hormone. This is further correlated with insulin sensitivity in a PWS cohort.^202^ It is proposed that increased overall fatty acid oxidation promoted by adiponectin signalling in skeletal muscle leads to increased insulin sensitivity, as previously stated.^209^ However, whether this is true for the pathophysiology of PWS remains unknown.

Our current understanding of the endocrine imbalance on the hypothalamic malfunction is still extremely poor. One of the biggest gaps to be filled in that sense is the “chicken‐or‐egg question” between the endocrine imbalance and the defective neuronal functioning. Is disrupted hypothalamic function the primordial cause of the obese phenotype, which is accompanied by endocrine alterations? Or is the defective endocrine production and/or secretion the main cause of the hypothalamic function?

## 6 HYPOTHALAMIC GLIAL CELLS AND INFLAMMATORY PATHWAYS IN PWS

The consumption of an obesogenic diet leads to structural and functional damage of the neuronal populations engaged with energy homeostasis regulation.^82^, ^87^, ^210^ The malfunction of those neurons is closely related to the activation of microglia cells.^83^, ^211^ Microglia are the resident immune cells of the CNS. The homeostatic functions of microglia are responsible for microenvironmental cleansing, mostly though phagocytosis of cellular debris and unwanted particles.^212^ This promotes a healthy microenvironment for optimal neuronal function throughout the CNS.^213^ Beyond the homeostatic functions of immunosurveillance, microglia play a central role in CNS pathologies, such as Alzheimer’s disease,^214^ Parkinson’s disease,^215^ multiple sclerosis^216^ and disorders in the CNS circuitry in control of energy homeostasis.^82^, ^83^, ^89^, ^217^, ^218^

Hypothalamic microglial activation towards a proinflammatory state is triggered by dietary composition and peripheral hormones.^218^ In the obese hypothalamus, activated microglia accumulate and produce neurotoxic and proinflammatory mediators, such as cytokines (mainly tumor necrosis factor; interleukin‐6; interleukin‐1 beta) and nitric oxide.^219^ Thus, the malfunction and numerical loss of INF neurons might be directly driven by microglial activation. Moreover, by looking into the post‐mortem hypothalamic tissues of type 2 diabetic patients, we have recently demonstrated that anti‐diabetic treatment is associated with a reduction of microglia cell number within the INF.^84^ This suggests that changes in microglial biology might also be implicated in the reversion of metabolic syndrome features. The obese phenotype is associated with a global inflammatory process that affects not only the hypothalamus.^220^ Resident and infiltrating immune cells trigger an inflammatory response in metabolic relevant organs, such as the liver, pancreas, and adipose tissue.^220^ Furthermore, both animal studies and preliminary evidence on human brain material demonstrate that inflammatory changes are first observed in the hypothalamus.^107^ Those alterations are also known to precede the peripheral metabolic disorders.

Currently, the role of inflammatory mediators in the pathophysiology of PWS is poorly explored and understood. Questions, such as whether the PWS patients have similar inflammatory outcomes as the non‐syndromic obese subjects, and whether there is any biological system that is differentially affected in either condition (i.e. is inflammation in the hypothalamus differently regulated in PWS and non‐genetic obesity?) still remain. Although plasma levels of cytokines are comparable among PWS and obese controls.^191^ The transcriptional signature of the hypothalami of PWS patients revealed a marked inflammatory process, concomitant with dampening of neuronal‐associated transcripts.^121^ The same study demonstrated reduced cell counts of astrocytes in PWS hypothalami. Astrocytes accumulate within the hypothalamus in conditions like obesity and T2DM^221^ and can contribute to the maintenance of an inflammatory process.^222^ This might suggest that the hypothalamic inflammatory‐associated changes found in PWS are more closely related to microglia than to other cell types, quite different from non‐genetic conditions. Beyond that, further studies are needed to find out whether the inflammatory changes in microglia are triggered by endocrine or dietary components, and whether the genes present in the PWS critical deletion region participate in the homeostatic or inflammatory changes of microglia.

Of importance, neuropeptides are known regulators of microglia function.^223^, ^224^, ^225^ The participation of neurotransmitters and neuropeptides as instructors of microglial function has gained crescent interest of the scientific community.^226^ However, the contribution of hypothalamic neuropeptides as immunomodulators is still poorly understood. It is also interesting that microglia cells comprehend a very heterogenous population in situ, and therefore it is difficult to translate the relevance of the in vitro findings into physiological terms.^227^ Therefore, the magnitude of the neuropeptidergic damping found in PWS hypothalami on the microglia biology is far to be fully understood. The two major antagonist neuropeptides expressed on the infundibular nucleus explored here (NPY and POMC) are known immunomodulators, especially in murine models. The NPY effects on microglia is broader explored in the retina, rather than in the hypothalamus.^228^ In brief, NPY is capable to inhibit LPS‐induced proinflammatory mediators (such as nitric oxide production and cytokines/interleukins) and has a suppressor effect on phagocytic capacity of microglia.^229^, ^230^ Likewise, the anorexigenic neuropeptides here mentioned have been broadly implicated in promoting an anti‐inflammatory phenotype. POMC‐derived peptides suppress LPS‐induced cytokine expression/secretion.^231^, ^232^ A similar pattern was found in other myeloid populations, as peripheral macrophages and neutrophils.^225^, ^233^, ^234^ Further research is necessary for the comprehension of the melanocortinergic system on non‐neuronal cells. Interestingly, among the neuropeptides addressed in this review, oxytocin is the one that has been further explored in microglial immunity. In primary microglia, oxytocin treatment leads to diminished production of LPS‐induced neurotoxic factors.^235^, ^236^ In concordance, intranasal oxytocin administration in adult mice limits microglial inflammatory response upon intraperitoneal LPS injection.^236^ It has been proposed that this phenotype is achieved through inhibition of signalling pathways classically associated with a proinflammatory response, such as the Nuclear Factor Kappa Beta and the mechanistic Target of Rapamycin.^237^, ^238^ Finally, the endocrine mediators explored here (leptin, ghrelin, and insulin) are also known regulators of microglia cells. In brief, it is broadly recognized that these hormones can modulate microglia cytokine production^239^, ^240^, ^241^ and phagocytic capacity.^89^, ^152^, ^242^ Therefore, the microglial biology might be a fundamental link between the molecular disruption of hypothalamic dysfunction and the clinical aspects of PWS.

## 7 CONCLUSION AND FUTURE PROSPECTIVE

Hypothalamic dysfunction is a hallmark of PWS. Defective hypothalamic neurocircuitry leads to the hyperphagic state, and consequently morbid obesity. This is translated into diminished expression of hypothalamic neuropeptides engaged in energy homeostasis regulation. Ultimately, there is a marked numerical loss of the neurons that compose both orexigenic (NPY‐expressing neurons) and anorexigenic populations (oxytocin‐expressing and presumably POMC‐expressing neurons). Furthermore, the neurocircuitry engaged in food intake and motivation are abnormally activated in PWS. Whether the disruption of the homeostatic control of eating behaviour is due to defective neuronal biology, an unhealthy microenvironment leading to neuronal dysfunction or primarily due to endocrine imbalance, is yet to be determined.

So far, symptomatic management of the disease is the only therapeutic option. The most successful approach in that sense is growth hormone therapy, which has been largely implicated in the attenuation of PWS related features (i.e. stature, body composition and motor and cognitive development). Despite the improvement of those symptoms growth hormone therapy fails to completely resolve the hyperphagia and obesity. It is also relevant to address that it is unknown to what extend improvement of hypothalamic dysfunction by growth hormone therapy is a consequence of its local signalling or overall amelioration of symptoms. Therefore, therapeutic approaches designed to specifically attenuate the hyperphagic state and obesity itself in PWS are necessary. Among the most promising agents in this respect are the glucagon‐like 1 receptor (GLP‐1R) agonists that have been extensively studied in obesity and T2DM and induce both weight loss and improved glycaemic control.^243^ Additional research is necessary to adopt GLP‐1R based therapies for PWS, but preliminary data show positive results. Salehi and collaborators reported that short‐term use of exenatide (a GLP‐1R agonist) promoted anorectic effects in a small PWS cohort (13‐25 years old).^244^ Importantly, the authors did not observe BMI changes during the short term administration and suggested further studies with long‐term administration as follow up, as well a combination of exenatide with behavioural modifications.^244^ Further, a more recent case report from Kim and colleagues demonstrated rapid and marked weight loss in a PWS adolescent (female, 18 years old) upon liraglutide (another GLP‐1R agonist) administration.^245^ Remarkably, the effects of GLP‐1R activation modulate hypothalamic neuronal populations engaged in energy homeostasis.^246^

Even though the therapeutic approach in PWS is still evolving, it is clear by now that hypothalamic dysfunction needs to be a key component in this equation regardless of the question whether it has a causal role. Overall, the effects of the temporal and spatial changes of hypothalamic neuronal and glial populations are not fully understood. Challenges such as the implications of the deletion at its full length rather than the role of each individual gene is one of the main obstacles in PWS field. Accumulating evidence indicates that neuroinflammation may be a major causative event on hypothalamic neuronal malfunctioning; and thus, it might be decisive in the design of new symptomatology management strategies. Further research is still necessary to comprehend the participation of glial cells, especially microglia, in the pathophysiology of PWS. Whether glial or neuronal cells are the first to be affected remains an open question at this moment.

## AUTHOR CONTRIBUTIONS

**Felipe Correa da SIlva:** Writing‐original draft; Writing‐review & editing. **Eric Fliers:** Supervision; Writing‐original draft; Writing‐review & editing. **Dick Swaab:** Supervision; Writing‐original draft; Writing‐review & editing. **Chun‐Xia Yi:** Conceptualization; Supervision; Writing‐original draft; Writing‐review & editing.

### PEER REVIEW

The peer review history for this article is available at https://publons.com/publon/10.1111/jne.12994.
